# Fine mapping and functional annotation of a QTL for resistance to tilapia lake virus in Nile tilapia (*Oreochromis niloticus*)

**DOI:** 10.1093/g3journal/jkaf276

**Published:** 2025-11-14

**Authors:** Agustin Barría, Pankaew Nunticha, Trịnh Quốc Trọng, Mahirah Mahmuddin, Carolina Peñaloza, Athina Papadopoulou, Ophelie Gervais, V Mohan Chadag, Ross D Houston, John A H Benzie, Diego Robledo

**Affiliations:** The Roslin Institute and Royal (Dick) School of Veterinary Studies, University of Edinburgh, Midlothian EH25 9RG, United Kingdom; Benchmark Genetics Norway AS, Bergen 5003, Norway; The Roslin Institute and Royal (Dick) School of Veterinary Studies, University of Edinburgh, Midlothian EH25 9RG, United Kingdom; Benchmark Genetics Norway AS, Bergen 5003, Norway; WorldFish, Jalan Batu Maung, Batu Maung, Bayan Lepas, Penang 11960, Malaysia; WorldFish, Jalan Batu Maung, Batu Maung, Bayan Lepas, Penang 11960, Malaysia; The Roslin Institute and Royal (Dick) School of Veterinary Studies, University of Edinburgh, Midlothian EH25 9RG, United Kingdom; Benchmark Genetics, Edinburgh Technopole, Penicuik EH26 0GB, United Kingdom; The Roslin Institute and Royal (Dick) School of Veterinary Studies, University of Edinburgh, Midlothian EH25 9RG, United Kingdom; The Roslin Institute and Royal (Dick) School of Veterinary Studies, University of Edinburgh, Midlothian EH25 9RG, United Kingdom; WorldFish, Jalan Batu Maung, Batu Maung, Bayan Lepas, Penang 11960, Malaysia; The Roslin Institute and Royal (Dick) School of Veterinary Studies, University of Edinburgh, Midlothian EH25 9RG, United Kingdom; Benchmark Genetics, Edinburgh Technopole, Penicuik EH26 0GB, United Kingdom; WorldFish, Jalan Batu Maung, Batu Maung, Bayan Lepas, Penang 11960, Malaysia; The Roslin Institute and Royal (Dick) School of Veterinary Studies, University of Edinburgh, Midlothian EH25 9RG, United Kingdom; Department of Zoology, Genetics and Physical Anthropology, Faculty of Biology, Universidade de Santiago de Compostela, Santiago de Compostela, 15705 Spain

**Keywords:** aquaculture, Nile tilapia, tilapia lake virus, genomics, fine mapping, selective breeding

## Abstract

Disease resistance is one of the main targets of animal breeding programs. In recent years, incorporating genomic information to accelerate genetic progress has become one of the priorities of the industry. Here, we combined population-scale whole-genome sequencing with differential gene expression and functional annotation analyses to study resistance to tilapia lake virus (TiLV) in a breeding Nile tilapia (*Oreochromis niloticus*) GIFT population. Fish with survival data from a natural TiLV outbreak were sampled and genotyped for 6.7 M SNPs using whole-genome resequencing and imputation. Our results confirmed a QTL located in the proximal end of *Oni22*, identifying 74 out of the top 99 markers associated with binary survival within a 10 Mb window. The marker explaining the highest genetic variance of TiLV resistance is located at 1.7 Mb and presents a substitution effect of 0.15. Additionally, other SNPs in several other chromosomes explained a high percentage of the genetic variance, with an important number located in 2 separate regions of *Oni09*. These results suggest an oligogenic architecture underlying resistance to TiLV, with several QTLs with moderate effect and many with small effect. Host transcriptomic analyses identified genes differentially expressed between resistant and susceptible genotypes according to the QTL in *Oni22*, highlighting *proteosome subunit beta type-9a*, and *ha1f* as potential causal genes. This is the first study combining whole-genome sequencing at a population scale with genomic approaches to assess the underlying genomic basis for TiLV resistance. Our results confirm and narrow down a QTL underlying this key trait in a major aquaculture species worldwide, and found novel QTLs in other chromosomes. The identified markers and genes have the potential to improve resistance to TiLV in Nile tilapia, significantly improving animal health and welfare.

## Introduction

With an average yearly growth rate of 6.7%, aquaculture is the fastest growing food production sector, dominated by Asia with 91.6% of total production in 2020 (FAO 2022a, b). Nile tilapia (*Oreochromis niloticus*) is among the most farmed finfish species worldwide, with a production of up to 4.4 M tons during 2020, representing 9% of total finfish aquaculture production (FAO 2022a, b). This species has been farmed since the 1980s, and several public and private breeding programs have been developed across Asia, America, and Africa, playing a key role in the food security and economic independence of many small rural communities in low- and middle-income countries.

Nonetheless, as in any other intensive production system, infectious diseases represent a significant threat to Nile tilapia's aquaculture production and sustainability. Tilapia lake virus (TiLV), a relatively recently identified RNA virus, has emerged as a major threat for tilapia farming, causing a systemic disease characterized by lethargy, loss of appetite, and respiratory distress (Turner et al. 2023), which can result in mass mortalities of up to 90% of the population (Dong et al. 2017; Fathi et al. 2017). This virus has shown both vertical and horizontal transmission (Eyngor et al. 2014; Jaemwimol et al. 2018; Dong et al. 2020), and although a few vaccines have been suggested to date (Mai et al. 2022; Yu et al. 2022), outbreaks tend to occur in early stages of production, which hinders the vaccination process (Thammatorn et al. 2019). Therefore, other preventive measures are preferred.

Genetic improvement for disease resistance offers a promising alternative to increase sustainability by preventing outbreaks and pathogen-related mortalities (Houston et al. 2020; Yáñez et al. 2022). Recent studies have shown the existence of substantial additive genetic variation for resistance to TiLV in a Nile tilapia breeding population, proving that resistance can be increased by selective breeding (Barría et al. 2020, 2021). Additionally, a QTL for resistance to TiLV has been identified in the proximal end of *Oni22*, highlighting the opportunity to improve this trait using marker-assisted selection (MAS) (Barria et al. 2021).

Identifying the causative genes and variants underlying this QTL can improve the efficiency of selection and enable downstream functional approaches. Barria et al. (2021) suggested several candidate genes for the TiLV resistance QTL in *Oni22*, including the *vacuolar protein-sorting-associated 52homolog* (*vps52*), *galectin 17* (*lgals17*), and *tripartite motif containing 29*. This was based on the use of a medium-density SNP array with ∼65 K SNPs (Penaloza et al. 2020).

Increased SNP densities are required to find the causative variants, which are unlikely to be present in the SNP array, and therefore enable the identification of the genes underlying key productive traits (Langridge et al. 2001). Whole-genome sequencing (WGS) can provide genotypes for all the genetic variants in the genome, allowing the identification of causative variants associated with important commercial traits. For instance, through a fine mapping analysis, Caceres et al. (2019) associated the *anti-Mullerian hormone* (*amh*) gene with sexual determination in GIFT-derived Nile tilapia populations—GIFT refers to “Genetically Improved Farmed Tilapia,” a Nile tilapia strain selectively bred for over 30 yr, mainly for growth (FAO 2022a, b). While generating population-level WGS can be considerably expensive, genotype imputation can substantially decrease the cost of obtaining whole-genome genotypes (Sinclair-Waters et al. 2020; Yoshida and Yanez 2021; Garcia et al. 2022; Sanchez-Roncancio et al. 2022).

Nonetheless, while WGS can increase the resolution of association analyses, due to linkage disequilibrium, it is not straightforward to discover the causative mutations and genes from genotypic data alone. The integration of genetic and transcriptomic data can further refine QTL mapping results, narrowing down the potential list of candidate genes and thereby improving the understanding of the underlying mechanisms (Pandit et al. 2010; Qin et al. 2020; Laoue et al. 2021; Gervais et al. 2022). For instance, through fine mapping analyses combining WGS, functional annotation, differential expression analyses, and CRISPR-Cas9, Pavelin et al. (2021) pointed to the gene *NEDD-8 activating enzyme 1* as the causative gene for the resistance to infectious pancreatic necrosis virus (IPNV) major QTL in Atlantic salmon (*Salmo salar*).

In this study, we combined a population-scale WGS and RNA-seq to reveal the genomic basis of resistance to TiLV in Nile tilapia. We have (i) generated whole-genome genotype data through imputation for a Nile tilapia population sampled after a natural TiLV outbreak, (ii) performed a high-scale genome-wide association analysis through a single-step genomic best linear unbiased prediction (ssGBLUp) approach to validate and refine the previous QTL region detected in *Oni22*, (iii) identified any potential novel QTLs underlying this trait, (iv) assessed the transcriptomic response during a TiLV infection, and (v) integrated genetic and transcriptomic data to evaluate whether expression differences in the genes in the QTL region could explain the QTL. Our results highlight several SNPs located in the proximal end of *Oni22* showing a highly significant association with TiLV resistance. Additionally, novel QTLs were detected in other chromosomes. A few potential candidate genes for the QTL in *Oni22* have been identified, including *proteosome subunit beta type-9a* (*psmb9a*), *vps52*, and *ha1f*, which might be modulating the host immune response.

These results will facilitate the improvement of resistance to TiLV through genomic-based selective breeding. Furthermore, the narrowed set of genes potentially underlying these QTLs will enhance our understanding of the functional mechanism involved in the resistance mechanism to this viral disease, offering new avenues for research, control, and treatment.

## Material and methods

### Fish population

The Nile tilapia population used in our study belongs to the GIFT strain, managed by WorldFish. The breeding population is established in Jitra Aquaculture Extension Center, Malaysia, and has been selected for a higher growth rate for 16 generations. The studied population (generation 16) comprises 124 families, generated by crossing 115 sires and 124 dams. For traceability, all fish were passive integrated transponder-tagged at approximately 110 d after hatching. Once the fish reached harvest weight (HW), they were transferred to a single pond. A TiLV outbreak was observed thereafter in this pond.

### TiLV outbreak

TiLV-related mortalities were collected daily throughout 14 d since the first mortality was observed. Once the mortality rates returned to a baseline, the remaining fish were euthanized through clove oil (400 mg/mL) and collected from days 15 to 19. Resistance to TiLV and phenotypic sex were measured for 1,821 fish. The former was defined as a binary trait (BS), with values 1 = fish survive/0 = fish die during the outbreak, and also as time to death (TD), that is, the number of days it took a fish to die after the first mortality was detected. Phenotypic sex was assessed by visual inspection. Clinical signs and necropsy associated with a TiLV infection process were observed throughout the collection period. The TiLV infection was later confirmed in a random sample of fish using RT-qPCR. The following forward and reverse primers were used: 5′–CTGAGCTAAAGAGGCAATATGGATT–3′, and 5′–CGTGCGTACTCGTTCAGTATAAGTTCT–3′, respectively, through an EvaGreen assay (Tattiyapong et al. 2018). For additional details about the phenotypic measures and the outbreak, please refer to Barria et al. (2020). Spleen and caudal fin were sampled from every fish for subsequent analyses. These were stored in RNAlater and ethanol 95%, respectively.

### DNA and RNA extraction

From the 1,821 animals collected during the outbreak, DNA was successfully extracted from 1,130 fish, which were successfully genotyped. The extracted DNA in the remaining 691 fish did not have enough quality required by the genotyping company. The Genomic DNA for most of the parents (*n* = 195) was also extracted. Therefore, a total of 1,325 fish were genotyped. Briefly, DNA was extracted following the protocol proposed by Aljanabi and Martinez (1997), with some modifications (Taslima et al. 2016). DNA integrity and purity were assessed in an agarose gel and in a Nanodrop by estimating the 260/280 and 260/230 ratios, respectively. DNA concentration was measured using a Qubit dsDNA BR assay kit (Invitrogen, Life Technologies).

Based on the most significant SNP for resistance to TiLV (Barria et al. 2021), fish were categorized as homozygous for the resistance allele (RR), heterozygous (RS), or homozygous for the susceptible allele (SS). The spleen RNA was extracted from 8 fish of each category (a total of 24 fish, 11 females and 13 males) from 24 different families. All of them were survivors of the natural outbreak and collected at day 15. A total of 50 to 70 ng of spleen was used to isolate RNA following a TRIzol (Invitrogen)—chloroform protocol. Briefly, samples were homogenized in a TissueLyser (Qiagen) for 9 s at 6 m/s, and mixed with 0.5 mL TRIzol and 0.1 mL of chloroform. After centrifugation, RNA was precipitated with 0.5 mL of isopropanol, and washed with ice-cold ethanol 75%.

Once the RNA was resuspended in 50 μL of nuclease-free water and incubated for 10 min at 60 °C, a purification step was done using the Direct-zol RNA purification kit (Zymo Research), which includes a DNase treatment. Finally, the RNA was eluted in 30 μL of RNAse-free water. RNA quality was measured in the Tapestation system (Agilent), whereas quantification was done by Qubit.

### 65 K SNPs genotyping

The genotyping pipeline and number of excluded animals throughout the QC steps is explained in detail in Barria et al. (2021). Briefly, each sample was genotyped with a Nile tilapia 65 K Axiom SNP array (Peñaloza et al. 2020). The raw data were filtered by call rate and dish quality control. Only “Polyhighresolution” markers were kept for further analyses. These markers were filtered using Plink v.1.09 (Purcell et al. 2007); only markers with call rate >0.95, MAF >0.05, and Hardy–Weinberg equilibrium (*P* > 1 × 10^−6^) were kept. Only fish with a call rate >95% were retained. A second filter, using trio information, was performed using Plink v.1.09, excluding markers and animals with an error rate ≥5%. The final dataset used for imputation comprises 47,915 SNPs identified in 950 TiLV-exposed fish and 162 of their parents.

### Whole-genome sequencing and SNP calling

A subset (*n* = 126) of the genotyped parents was whole-genome sequenced. These fish represent the parents of 67 of the TiLV-exposed families (567 out of 950 fish), with 65 sires and 61 dams. Extracted DNA was sequenced on an Illumina Novaseq 6000 platform as 150 bp paired-end reads. Read quality was assessed by using FastQC v.0.11.9, and raw reads were filtered using Trimgalore v0.6.3; adaptors were trimmed, and bases with a Phred score <20 were removed. The remaining high-quality reads were aligned to the Nile tilapia reference genome (Genbank accession number GCF_001858045.3; Conte et al. 2017) using Burrows–Wheeler aligner v0.7.17 (Li and Durbin 2009). The sequence coverage was estimated using Picard v.2.25.4, ranging from 7.7× to 16.1×, with an average of 12.0×.

SNP variants were called using BCFtools v.1.9 (Li 2011). The indels, non-biallelic markers, and SNPs within 10 bp of an indel were discarded. Then, only markers with mapping quality >30, SNP base quality >300, raw read depth between 2,000 and 3,500, and located in chromosomes were kept. After quality control, 7,271,637 SNPs in 126 parental fish were retained for further analysis.

### Data imputation

The fish collected during the TiLV outbreak (*n* = 950) with high-quality genotypes (47 K SNPs), representing generation 16 of the WorldFish breeding program, were imputed to the >7 M SNPs discovered in their parents (Generation 15th). The imputation was performed using AlphaImpute2 (Antolin et al. 2017; Whalen and Hickey 2020) with default parameters. A deep pedigree record, containing information up to the base population, was provided for imputation. To obtain an estimate of imputation accuracy, the SNPs in the array were grouped according to their relative position (ie, group 1: SNPs 1, 11, 21, 31, …; group 2: SNPs 2, 12, 22, 32, …; etc.). Then, the genotypes of the SNPs in each group were masked separately, and imputation was performed for each group. Imputation accuracy was then calculated as the correlation between the imputed genotypes and the real genotypes.

Finally, all markers with a MAF <0.01 were removed (*n* = 570,303). Thus, a total of 6,701,334 SNPs were considered for further analyses.

### Estimation of variance components

To estimate genetic variance parameters for both resistance traits, a linear univariate animal model was fitted for TD, whereas a threshold animal model was used for BS. For both traits, an ssGBLUP (Wang et al. 2012) was implemented through the BLUPF90 family programs (Lourenco et al. 2022).

The model was fitted as follows:


y=μ+Xb+Za+e

where *y* represents the phenotype (BS or TD), μ is the population mean, the vector **b** contains sex as fixed effect, and weight and age at harvest as covariates. The effect of these factors was significant (*P* < 0.05) for both traits. The vector of the additive genetic effects is represented by a, whereas X and Z are the incidences matrices. The following distributions were assumed: $a∼N(0,H\sigma_{a}^{2}$**)** and $e∼N(0,I\sigma_{e}^{2}$**)**, where $\sigma_{a}^{2}$ and $\sigma_{e}^{2}$ are the additive genetic and residual variance, respectively, H is the kinship matrix (Aguilar et al. 2010) and I is the identity matrix. The inverse of the **H** matrix, combining pedigree and genotype relationship coefficients, is defined as follows:


$$H^{-1}=\;A^{-1}+\;\left[\begin{array}{cc} 0 & 0\\ 0 & G^{-1}-\;A_{22}^{-1} \end{array}\right]$$

where $A_{22}^{-1}$ and $G^{-1}\;$ represent the inverse of the pedigree-based relationship matrix for the genotyped animals, and the inverse of the genomic relationship matrix, respectively. Thus, the genomic information of the approximately 6.7M SNPs for the 950 fish was combined with slightly more than 86 K relationship records from the pedigree, up to the base population.

The variance parameters for BS were estimated through a Bayesian framework using *gibbsf90+* available in the BLUPF90 software (Lourenco et al. 2022). The Gibbs sampling scheme consisted of 2,000,000 iterations, discarding the initial 200,000 iterations. From the remaining 1,800,000 iterations, 1 sample every 50 iterations was retained, yielding a total of 36,000 independent samples for genetic analyses. Convergence was evaluated by the R package CODA (Plummer et al. 2006).

For the genetic correlation between both resistance traits, an ssGBLUP approach was used, fitting the same model described earlier. The BS trait was treated as a linear trait in this case. When estimating the genetic correlation of both BS and TD with HW, age and sex were included as fixed effects for HW.

### Genome-wide association analysis and SNP effect

The initial set of 7 M imputed SNPs was filtered to retain only those with an MAF >0.01, resulting in a total of 6,701,334 SNPs. The GWAS was conducted for BS and TD using a single-step GBLUP, implemented via the BLUPF90 software. A sliding window variance method, with a window size of 1 SNP (*n* = 1), was employed to estimate the proportion of genetic variance explained by each individual SNP. The linear model used for this analysis was identical to the one shown earlier. More details can be found in Barria et al. (2021). Considering AA, AB, and BB as the predicted values for each genotype, the additive (*a*) effect was estimated as *a* = (AA – BB)/2.

### Transcriptomic analyses

RNA-seq polyA enrichment libraries were generated and sequenced at Novogene (Cambridge, United Kingdom) using a Novaseq 6000 as 150 bp paired-end reads. Raw reads were quality checked and trimmed using FastQC v0.11.9 and trimgalore v0.6.3, respectively, whereas adapters were cut with cutadapt v1.9.1 (Marcel 2011). Bases with a quality <30 and reads with length <20 were discarded, obtaining an average of 23,373,408 reads per sample (SD = 2,148,848). Kallisto v0.44.0 (Bray et al. 2016) was used to pseudoalign the high-quality trimmed reads against the Nile tilapia reference transcriptome (Genbank accession number GCF_001858045.3; Conte et al. 2017) to estimate the expression of all transcripts. Output was then summarized to the gene level using tximport (Soneson et al. 2015). Only genes with a minimum of 10 counts and found in at least 3 samples were considered for further analyses. Thus, a total remaining of 19,941 genes were tested for DE using DESeq2 (Love et al. 2014). Genes with an adjusted false discovery rate *P*-value <0.05 were categorized as DE.

## Results

### Field outbreak

As previously reported (Barría et al. 2020; Barria et al. 2021), a field outbreak in a GIFT Nile tilapia population (generation 16) was characterized by both necropsy and clinical signs typically associated with TiLV infections. RT-qPCR confirmed the presence of TiLV in half of the surviving fish and in 100% of the dead fish tested. The population consisted of 1,821 fish, and the cumulative mortality during the field outbreak reached 39.6%. For further details regarding this natural field outbreak, please refer to Barría et al. (2020) and Barria et al. (2021).

### Estimation of variance components and heritability

The whole population was imputed to whole-genome high-quality genotypes (7.21M SNPs—estimated imputation accuracy 89.8 ± 2.9). A posterior QC removed almost 600 K markers with an MAF <0.01, and thus approximately 6.70 M imputed were kept for further analyses. A moderate heritability estimate was obtained for TD (0.26 ± 0.05), while estimations for binary survival (BS) were higher, with a value of 0.65 ± 0.05 (Table 1). The genetic correlation coefficient between the 2 estimates of resistance to TiLV was 0.95 ± 0.02, indicating that there is a single underlying trait. The genetic correlation between resistance to TiLV and HW was not significant, with estimates of 0.14 ± 0.10 and 0.14 ± 0.12 for BS and TD, respectively.

**Table 1.** Estimates of variance components ± standard error for host resistance to TiLV in a breeding Nile tilapia (*Oreochromis niloticus*) population estimated by single-step GBLUP (ssGBLUP).

Parameters^a^	BS^b^	TD^b^
$\sigma_{a}^{2}$	1.98 ± 0.48	9.12 ± 1.88
$\sigma_{e}^{2}$	1.01 ± 0.05^c^	26.18 ± 1.37
$h^{2}$	0.65 ± 0.05	0.26 ± 0.05
$r_{g}$	0.98 ± 0.02	

^a^ $\sigma_{a}^{2}$ , additive genetic variance; $\sigma_{e}^{2}$, residual variance; $h^{2}$, narrow-sense heritability; $r_{g}$, genetic correlation.

^b^BS, binary survival; TD, time to death.

^c^Residual variance for BS was set to 1.

### Genome-wide association analysis

**Fig. 1.**
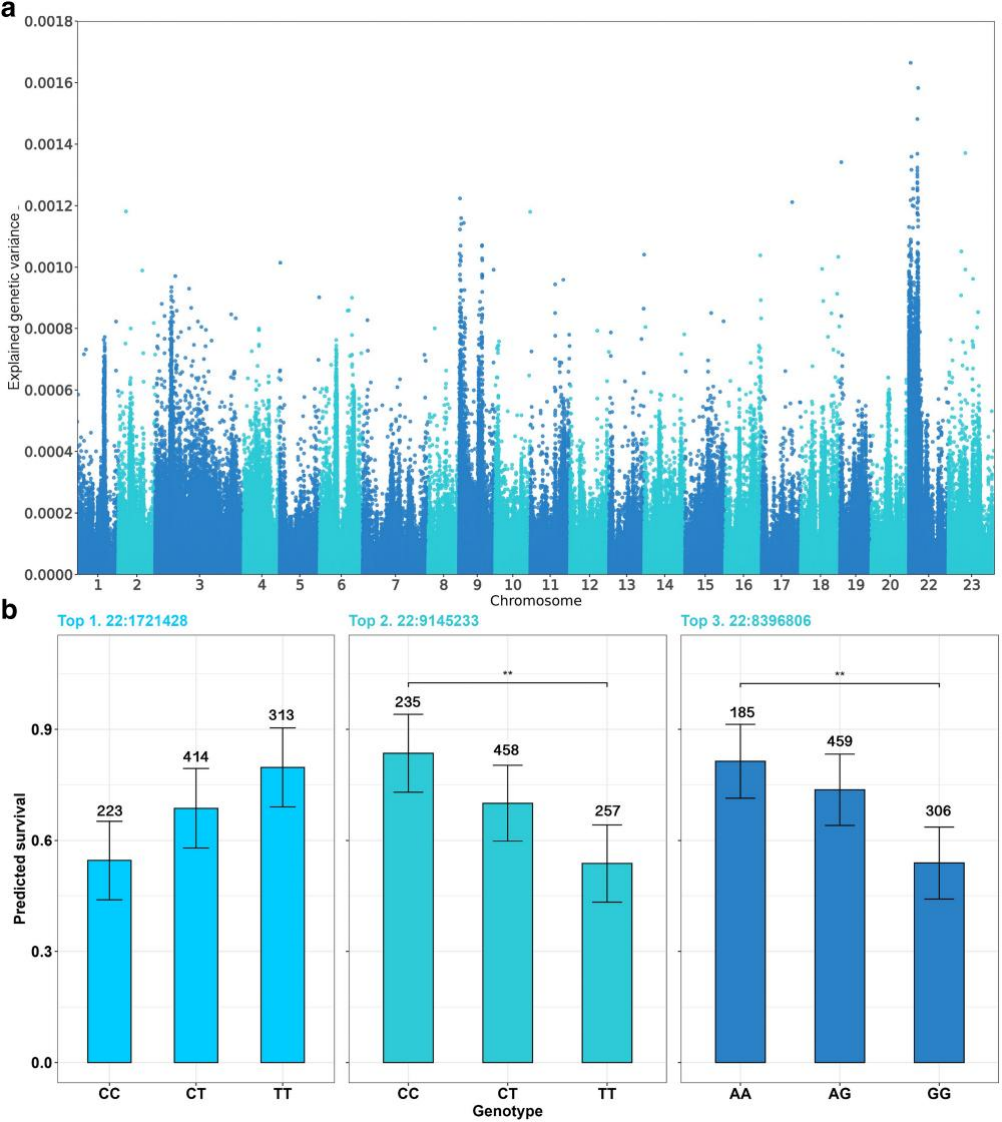
Manhattan plot and predicted survival rate for resistance to tilapia lake virus in a GIFT Nile tilapia (*Oreochromis niloticus*) breeding population. Manhattan plot of GWAS for host resistance to TiLV as a BS (a). Host resistance predictive survival proportion for the genotypes of the top three markers. Numbers above the bars indicate the number of fish with the specific genotype, while the bars represent the standard error (b). Asterisks show statistical significance from pairwise *t*-test comparing predicted survival values across genotypes.

A GWAS for TD identified 31 markers exceeding a genetic variance explained threshold of 0.0010. Of these, 26 markers (84%) are in *Oni22*, within a 10-megabase (Mb) window (Supplementary Fig. 1). Notably, 15 of them cluster within a 170 Kb window size. The remaining 5 markers are distributed across *Oni03*, *Oni17*, *Oni18*, and *Oni09*, with 2 markers. For BS, 99 markers exceed the same threshold of genetic variance (Fig. 1a). Of these, 74 (75%) are located in *Oni22*, spanning an approximately 10 Mb window, ranging from 125,207 to 9,335,256, with a notable concentration of 48 markers within a 1.1 Mb window. *Oni09* harbors 15 markers above the threshold, located in 2 different regions, with 11 clustered within a 4 Mb window at the proximal end of the chromosome (Fig. 1a). Lastly, a few other markers of interest were found in *Oni02*, *Oni05*, *Oni10*, *Oni113*, *Oni16*, *Oni17*, *Oni18*, *Oni19*, and *Oni23*, with 1 marker each.

The top 3 SNPs associated with BS showed a maximum average allele substitution effect of 0.15 in the probability of survival to TiLV (Table 2). For instance, for the marker explaining the highest genetic variance, the predicted average survival of fish carrying both resistance alleles reached up to 0.80, whereas the survival of fish homozygous for the susceptibility allele is 0.55 (Fig. 1b).

**Table 2.** Summary statistics for top three markers for host resistance to TiLV.

SNP	BP^a^	GenVar	*a* ^b^	*P*val (*a*)^c^
1	1,721,428	0.001664	0.125	1.3E−08
2	9,145,233	0.001583	0.149	3.1E−10
3	8,396,806	0.001482	0.137	4.4E−09

^a^Position in the Nile tilapia reference genome.

^b^Average effect of allele substitution.

^c^ *P-*value of the Student's *t*-test distribution for the allele substitution effect.

From the total set of markers for TD and BS found above the threshold, 26 of them were shared, and 22 of these are found in *Oni22* (Table 3), highlighting the key role of this chromosome in the resistance to TiLV. The frequency of the top 10 common markers can be found in Table 3, ranging from 0.431 to 0.488

**Table 3.** Top 10 makers in common associated to TiLV resistance defined as time to death and binary survival in a breeding Nile tilapia (*Oreochromis nilotocus*) population.

SNP	BP^a^	GenVar TD	GenVar BS	MAF
1	1,721,428	1.59E−03	0.001664	0.4526
2	9,145,233	1.34E−03	0.001583	0.4884
3	8,396,806	1.25E−03	0.001482	0.4363
4	8,396,773	1.13E−03	0.001369	0.4358
5	2,539,310	1.17E−03	0.001359	0.4321
6	8,394,308	1.24E−03	0.001324	0.4663
7	2,539,659	1.12E−03	0.001317	0.4421
8	8,359,021	1.00E−03	0.001314	0.4679
9	8,360,101	1.08E−03	0.001302	0.4316
10	8,394,869	1.06E−03	0.001294	0.4311

^a^Position in the Nile tilapia reference genome.

### Fine mapping and candidate genes

**Fig. 2.**
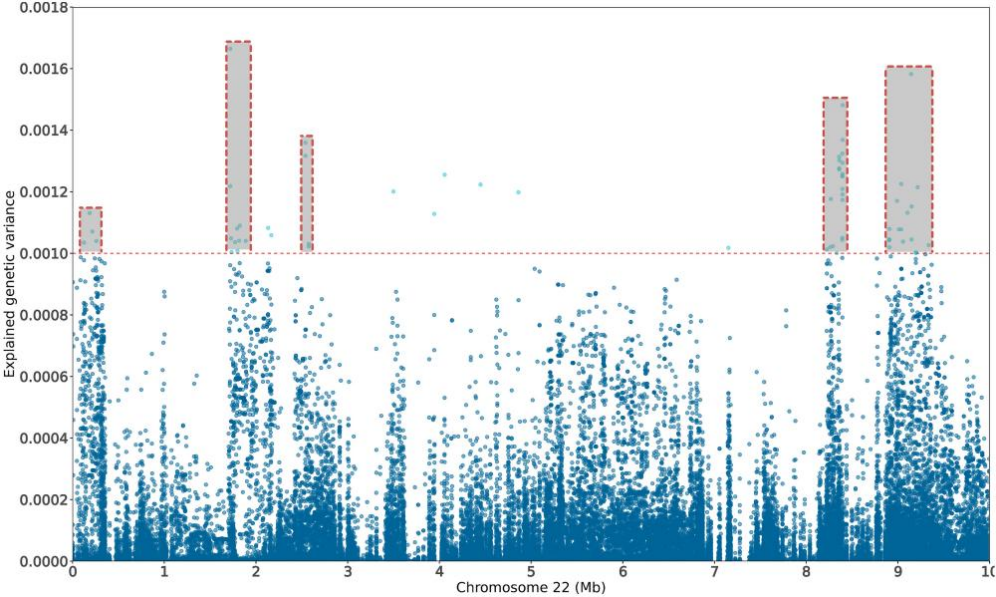
Fine mapping of the genomic regions associated with resistance to tilapia lake virus in a breeding Nile tilapia (*Oreochromis niloticus*) population. Manhattan plot for resistance to TiLV showing the first 10 Mb of *Oni22*.

Closer examination of the QTL region located in *Oni22* revealed the presence of 5 main groups of markers associated to BS across the 10 Mb window (Fig. 2a). Of the 74 markers above the threshold, 21 (28%) are clustered within a 171 Kb window (8,225,465 to 8,396,806), with 6 of the top 10 markers in terms of explained genetic variance located in just 37 Kb, ranging from 8,359,021 to 8,396,806. Additionally, the proximal end of *Oni22* contains the marker explaining the highest genetic variance for BS, in position 1,721,428, clustering with 9 additional markers of interest.

Only genes located within the 5 different groups were evaluated for their potential role in the host response to TiLV. Notably, *vps52* lies between 2 markers located in group 1, at approximately 22 Kbp. In the same group, *class I histocompatibility antigen*, *F10 alpha chain* (*ha1f*), and *E3 Ubiquitin-Protein ligase* (*ring2*; also called *rnf2*) were also found. All of them have been previously associated with the modulation of the host immune response during the viral infection process. Additionally, the *psmb9a* and *pvr cell adhesion molecule (pvrl2l)*, located in group 4 and group 5, respectively, are known for their antiviral role during infections. The full list of genes surrounding the leading SNP within each peak is shown in Table 4.

**Table 4.** Genes of interest within each peak associated to host resistance to TiLV defined as BS.

Group	SNPs in QTL	QTL region Size (bp)		Gene names
		Initial position	Final position	
1	4	125,207	259,543	*ring2, rps18, vps52, ptk7, hmcn1, ha1f, lgals17, h2-k1*
2	10	1,719,784	1,888,302	*Grik5, oni-mir-10903*
3	6	2,132,756	2,576,678	*pomca, ndufb9, tatdn1, rnf139, tmem65, mtss1, scgn, enpp2*
4	21	8,225,465	8,396,806	*rxrba, col11a2, brd2a, tap2a, psmb9a*
5	27	8,903,428	9,335,256	*flot1a, tcf19l, pvrl2l*

### Nile tilapia response to TiLV infection

**Fig. 3.**
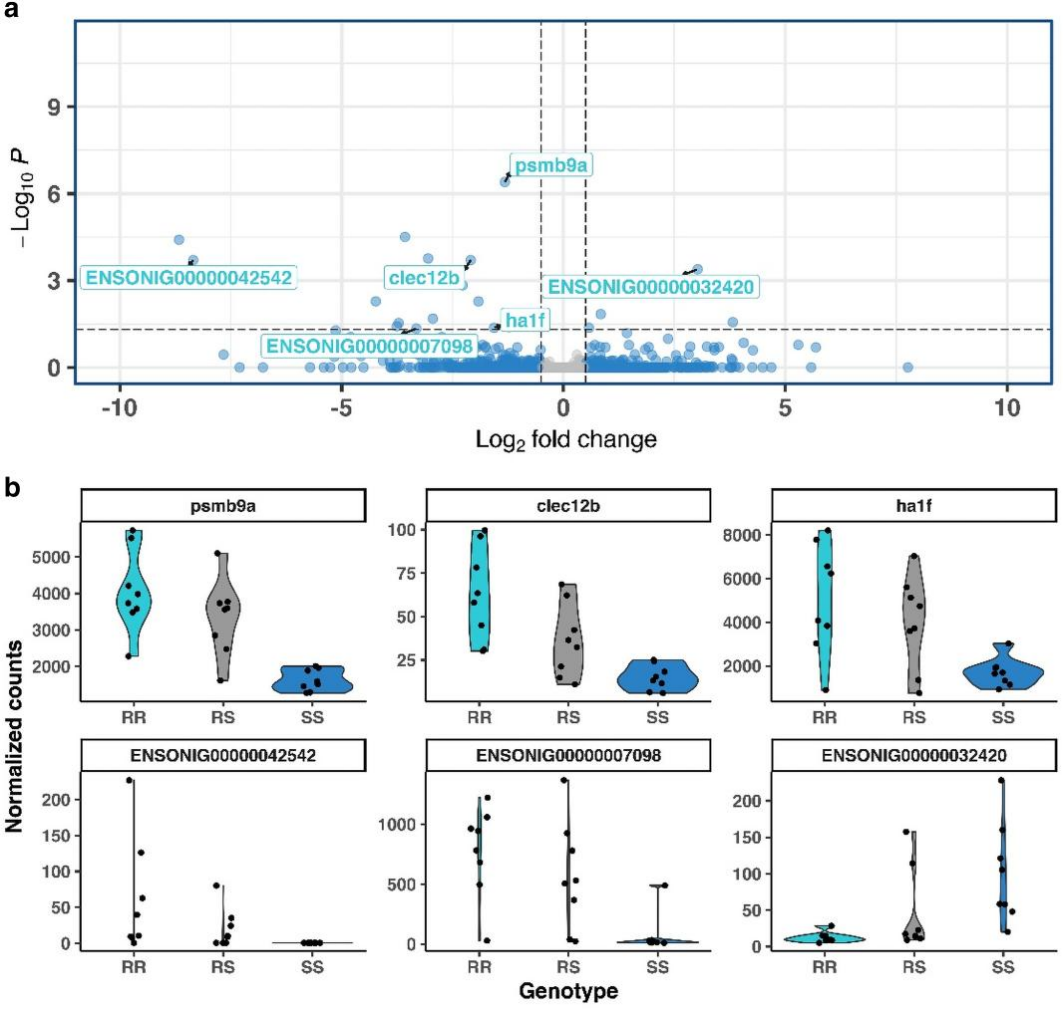
Host transcriptomic response during recovery phase after a TiLV infection. Volcano plot highlights the DE genes located in *Oni22*, the chromosome containing the QTL for resistance to TiLV (a). Comparison of the normalized counts for the 6 genes located within the 10 Mb QTL depending on the fish genotype for the top SNP for resistance to TiLV (b).

The spleen transcriptomes of *RR*, *RS*, and *SS* animals according to the TiLV resistance *Oni22* QTL genotype were compared during the recovery phase after the TiLV natural outbreak. A total of 21 genes showed DE between genotypes (Fig. 3a). The chromosome containing the QTL in *Oni22* contributed with the highest number of DE genes (*n* = 6), all located within the 10 Mb QTL region, including *C-type lectin domain family 12 member* (*clec12b*), *psmb9a*, and *ha1f*, highlighted in Fig. 3a. *Ha1f* is located at 300 Kb, in the region showing the maximum association with resistance to TiLV*, clec12b* is found at 1.0 Mb, and *psmb9a* is found at 8.3 Mbp, the region with the most significant markers. The 3 remaining genes are unannotated (*ENSONIT00000051186 at* 5.0 Mb, *ENSONIG00000042542* at 4.5 Mb, and *ENSONIG00000007098* at 7.8 Mb).

The differences between genotypes for the 6 DE genes in *Oni22* are shown in Fig. 3b. For 5 of these genes, including *ha1f*, *clec12b*, and *psmb9a*, fish with 2 resistant alleles have higher expression, while the lowest expression is found in fish carrying 2 susceptible alleles. *Psmb9a* showed the most significant differences between RR and SS fish.

## Discussion

In previous studies, we have shown the existence of significant additive genetic variation for host resistance to TiLV in a GIFT Nile tilapia population using both pedigree (Barría et al. 2020) and genomic data from a ∼65 K SNP array (Barria et al. 2021). Using the same phenotypic data collected from a natural field outbreak, and described in the mentioned studies, we have now conducted a fine mapping analysis using 6.7 M imputed SNPs after applying population-scale WGS. Thus, we significantly increased the marker density to refine and increase the accuracy of the previously discovered QTL region, but also identified new genomic regions associated with host resistance to TiLV. Furthermore, we have integrated genetic and transcriptomic information by comparing the spleen transcriptome between fish with different TiLV resistance QTL genotypes, determined based on the most significant marker previously described in Barria et al. (2021), and therefore identified potential functional candidate genes underlying the resistance trait.

### Whole-genome sequencing improves the accuracy of genetic estimation

A high proportion of the observed phenotypic variance for both definitions of TiLV resistance (BS and TD) is due to additive genetic variation in the studied Nile tilapia population. Significant heritabilities of 0.65 and 0.26 were estimated for BS and TD, respectively, which are in agreement with those estimated previously using pedigree and SNP array (Barría et al. 2020, 2021), of 0.63 and 0.69, respectively. Still, this estimate is more accurate than previous studies since we have combined pedigree data and high-density genomic data. The latter captures a larger proportion of the genetic variation across the genome and therefore represents a more comprehensive assessment of the contribution of the genetic factors for resistance to TiLV, especially if the new variants are in LD with rare causal variants (Yang et al. 2015; Al Kalaldeh et al. 2019; Wainschtein et al. 2022). The current estimates are higher than the usual heritability estimations for disease resistance in aquaculture species (Yanez et al. 2015), indicating a high potential to improve this trait by selective breeding.

In agreement with previous estimations, our results reveal no significant genetic correlation between resistance to TiLV and HW, indicating the potential to improve both key traits simultaneously. Previous studies in other species have shown varying genetic correlations between growth and disease resistance to various viruses. For instance, in European sea bass (*Dicentrarchus labrax),* a significant and negative genetic correlation of −0.35 ± 0.14 was estimated between body weight and resistance to viral nervous necrosis (VNN) (Doan et al. 2017), while in salmonid species, both positive and nonsignificant genetic correlations have been estimated between resistance to IPNV and HW (Lillehammer et al. 2013; Flores-Mara et al. 2017). Similar results have been observed for bacterial diseases (Yáñez et al. 2016; Xiong et al. 2017; Barria et al. 2018, 2019). In sum, there is no consistency in the genetic relationship between growth and disease resistance traits, as this is likely dependent on the biological mechanism underpinning disease resistance.

### Marker-assisted selection can lead to fast gains in resistance to TiLV

The size of the mapped QTL is encouraging for MAS and can substantially improve TiLV resistance in Nile tilapia breeding populations, as there is a difference in survival rate between opposite homozygous fish ranging from 24% to 30%. For markers on which the minor allele is the one conferring the resistant phenotype, there is ample capacity for improvement via selective breeding.

The success and application of MAS in aquaculture breeding have already been demonstrated with the selection of fish carrying alleles conferring resistance to IPNV (Houston et al. 2008; Moen et al. 2009; Houston et al. 2010), significantly reducing the related mortalities in Norwegian Atlantic salmon (Sommerset et al. 2021). Although the current QTL explains less genetic variation, it still highlights the potential of genetics to enhance key commercial traits in farmed aquaculture species. Additionally, combining this information with candidate genes underlying the trait can lead to alternative strategies to reduce TiLV-related mortalities, for example, via the application of CRISPR-cas9 editing (Gratacap et al. 2019).

### A QTL for resistance to TiLV in the proximal region of chromosome 22 in the context of an oligemic architecture

Disease resistance traits are frequently polygenic (Fraslin et al. 2020). However, some exceptions have been described in aquaculture species, where major or moderate QTLs have been found, specifically for viral diseases. For instance, Vallejo et al. (2019) found 10 QTLs with moderate effect for resistance to the infectious hematopoietic necrosis virus (IHNV) in rainbow trout, while Gonen et al. (2015) found a major QTL associated with resistance to salmonid alphavirus in chromosome 3 of Atlantic salmon. In this same species, and by using data collected from outbreaks, Houston et al. (2008) and Boison et al. (2019) identified major QTLs for resistance to IPNV and piscine myocarditis virus, respectively.

The current study used almost 1,000 fish, and imputation increased the number of markers more than 100× compared to our previous study, from around 47 K to 6.7 M SNPs (Barria et al. 2021), representing 1 SNP every 148 bp. This increase in SNP density allowed us to more precisely identify the genomic variants associated with resistance to TiLV, refining the position of the QTL located in the proximal end of *Oni22* (Barria et al. 2021) and narrowing down the potential causative genes underlying the trait. The highest associated marker is located at 1,721,428 bp in *Oni22*, which was found highly associated with resistance as BS and as TD. Previously, Barria et al. (2021), found the most significant marker at 255,104 bp in the same chromosome. Although the 2 approaches were slightly different (GBLUP vs ssGBLUP), and Barria et al. (2021) provided *P*-values rather than genetic variance, a cluster of markers within this region was also found to be associated with resistance to TiLV in this study.

Consistent with Barria et al. (2021), our study also identified a QTL associated with resistance to TiLV in *Oni03*. Additionally, potential novel QTLs were found, including one in *Oni01* and two in *Oni09*. The discovery of these QTLs is likely the result of increased marker density, but also of the enhanced power of the ssGBLUP approach, achieved by combining information from animals with and without available imputed genotypes, increasing the number of samples from 950 to 1,821. These findings indicate that Oni09 plays a significant but secondary role compared to Oni22, with *Oni01* and *Oni03* also having a small impact on the trait, strongly suggesting an oligogenic architecture for host resistance to TiLV.

Nonetheless, we cannot discard the possibility that other minor QTL exist for this trait, as previously proposed (Vallejo et al. 2019; Sinclair-Waters et al. 2020; Barria et al. 2021). Further experimental challenges in different conditions or different breeding populations need to be performed to confirm the presence or absence of additional QTL. For instance, typical controlled experimental challenges based on intraperitoneal (IP) injections might result in different resistance mechanisms and, therefore, different associated genomic regions.

### *Vps52*, *lgals17*, and *ha1f* are clear positional candidates potentially underpinning the QTL in *Oni22*

Given the low LD in this population (Penaloza et al. 2020), combined with the high SNP density, we were able to narrow down the candidate genes to those adjacent to the most significant SNPs. This approach helped us avoid the investigation of large genomic windows harboring genes that are probably weakly or not associated with resistance to TiLV. As a result, while we highlighted a smaller set of genes when compared to Barria et al. (2021), we increased the likelihood that these genes are functionally involved in resistance to TiLV.

Encouragingly, genes previously reported to be involved in the host immune response during viral infections colocalized with the narrowed QTL region. For instance, we found *vps52* flanking markers in cluster 1, consistent with the results of Barria et al. (2021). This gene is part of the Golgi-associated retrograde protein complex, involved in transport from endosome to trans-Golgi (Conibear and Stevens 2000; Liewen et al. 2005). However, it also facilitates the wrapping of intracellular virions, modulating viral infections by reducing viral replication (Harrison et al. 2016; Realegeno et al. 2017).

Another gene located in the same cluster 1 is *ha1f,* encoding a specific type of major histocompatibility complex class I. Found in the cell surface and containing transmembrane heavy chain molecules (Grimholt et al. 2019), it plays a key role in controlling pathogens by presenting antigens to the cell defense system (Hansen and La Patra 2002; Wieczorek et al. 2017). Similarly, to our study, this gene was found in close association with the QTL identified for resistance to PMCV in Atlantic salmon (Boison et al. 2019) and suggested as a key player in the resistance trait.

In Barria et al. (2021), the most significant marker was located within the second intron of *lgals17*, a gene with pattern recognition receptor functions (Vasta 2009), which is also located in cluster 1. Members of this family have already been associated with antiviral activity in other fish species. For example, upregulation of this gene has been observed during infectious salmon anemia virus (ISAV) and viral hemorrhagic septicemia virus infections in salmonids (O'Farrell et al. 2002; Jorgensen et al. 2008) and during an infection with VNN in European sea bass (Poisa-Beiro et al. 2009).

### Differentially expressed genes in Oni22

The transcriptomic study aimed to discover potential differences in gene expression between fish with different QTL genotypes, selected based on the SNP most significantly associated with resistance to TiLV (Barria et al. 2021). The transcriptomic analysis revealed various DE genes in the region with an antiviral role, potentially highly transcribed during the infection. The most DE gene was *psmb9a*, with fish carrying both copies of the allele conferring resistance showing the highest levels of transcription. Interestingly, this gene is located in the cluster with the largest number of markers associated with resistance to TiLV. This gene is involved in the generation of peptides needed for the correct presentation of antigens by class Ia molecules (Boes et al. 1994; Kuckelkorn et al. 1995). A high upregulation of this gene was found during an acute IHNV-related infection in the spleen of rainbow trout (Hansen and La Patra 2002). The 2 times higher expression of *psmb9a* in resistant fish again suggests the activation of cellular immunity during TiLV infection, as previously observed for piscine orthoreovirus in Atlantic salmon (Vallejos-Vidal et al. 2022), and a potential role of antigen-presenting pathways in the resistant phenotype.

The *ha1f* gene is located in the proximal end of *Oni22*, whereas the remaining genes are found within the 10 Mb QTL region. In previous studies, *ha1f* has been found to be overexpressed during an ISAV infection in Atlantic salmon head kidney, modulating the host adaptive immune response (Valenzuela-Miranda et al. 2015). This gene was also found highly upregulated in Nile tilapia spleen 24 and 120 h post an IP TiLV challenge (Wang et al. 2020). The high expression of this gene in the *RR* fish compared with the *SS* in our study highlights the likely key role of the antigen-presenting molecules during TiLV infection.

## Conclusions

The incorporation of WGS provides new insights into the genomic architecture of resistance to TiLV in Nile tilapia. The higher SNP density allowed narrowing the QTL to the proximal end of *Oni22* and discovering novel QTLs in other chromosomes, suggesting an oligemic architecture underlying host resistance to TiLV. *psmb9a* and *ha1f* represent strong candidate genes potentially underlying the *Oni22* TiLV resistance QTL. This knowledge will be crucial in guiding further studies on TiLV resistance, including potential functional studies to generate fully resistant fish via genome editing. Ultimately, this information will have key implications for the development of effective therapies for TiLV in Nile tilapia, contributing decisively to food security and animal welfare.

## Supplementary Material

jkaf276_Supplementary_Data

## Data Availability

SNP array data are available in Peñaloza et al. (2020) . The raw WGS data of the 126 fish and the RNAseq data supporting this study have been submitted into the BioProject Database and can be accessed with ID: PRJNA1235501 and ID PRJNA1238277, respectively. Phenotypic and genotypic data can be found in the Figshare repository (10.6084/m9.figshare.28806155). Supplementary Figure 1 shows the Manhattan plot for TD. Supplemental material is available at G3 online.
