# Supplementary material for: Fine mapping and functional annotation of a QTL for resistance to tilapia lake virus in Nile tilapia (Oreochromis niloticus)
Source: G3 (Bethesda). 2025 Nov 14;16(1):jkaf276. doi: 10.1093/g3journal/jkaf276 (PMC12774591; doi:10.1093/g3journal/jkaf276)
Supplement: jkaf276_Supplementary_Data [file jkaf276_supplementary_data.docx]

**Figure S1. Manhattan plot for time to death to Tilapia Lake Virus (TiLV) in a GIFT Nile tilapia (*Oreochromis niloticus*) breeding population.** Manhattan plot of GWAS for host resistance to TiLV as time to death (TD).
